# Beyond Tradition: Exploring Cutting-Edge Approaches for Accurate Diagnosis of Human Filariasis

**DOI:** 10.3390/pathogens13060447

**Published:** 2024-05-24

**Authors:** Damian Pietrzak, Julia Weronika Łuczak, Marcin Wiśniewski

**Affiliations:** 1 Division of Parasitology and Parasitic Diseases, Department of Preclinical Sciences, Institute of Veterinary Medicine, Warsaw University of Life Sciences—SGGW, 02-786 Warsaw, Poland; damianpietrzak23@gmail.com; 2 Faculty of Animal Breeding, Bioengineering and Conservation, Warsaw University of Life Sciences—SGGW, 02-786 Warsaw, Poland; luczak.ju@icloud.com

**Keywords:** filariasis, parasitology, Nematoda, molecular biology techniques, immunoenzymatic tests, diagnostics

## Abstract

Filariasis is recognised as a global public health threat, particularly in tropical and subtropical regions. It is caused by infection with a nematode parasite of the superfamily Filarioidea, including *Wuchereria bancrofti*, *Brugia malayi*, *Onchocerca volvulus*, and *Onchocerca lupi*. Three main types of filariasis have been classified: lymphatic filariasis, subcutaneous filariasis, and serous cavity filariasis. The symptoms exhibited by individuals afflicted with filariasis are diverse and contingent upon several variables, including the species of parasite, the host’s health and immune response, and the stage of infection. While many classical parasitological techniques are considered indispensable tools for the diagnosis of parasitic infections in humans, alternative methods are being sought due to their limitations. Novel tests based on host–parasite interactions offer a rapid, simple, sensitive, and specific diagnostic tool in comparison to traditional parasitological methods. This article presents methods developed in the 21st century for the diagnosis of filariasis caused by invasion from *W. bancrofti*, *B. malayi*, *O. volvulus*, and *O. lupi*, as well as techniques that are currently in use. The development of modern diagnostic methods based on molecular biology constitutes a significant advancement in the fight against filariasis.

## 1. Introduction

Filariasis is a disease caused by parasites belonging to the phylum Nematoda, which is the third most species-rich in the animal kingdom [1,2]. It is estimated that there may be as many as 24,000 species of nematodes parasitizing vertebrates, the majority of which remain undescribed [3,4,5]. Filariasis of animals, especially mammals, can be transmitted to humans and is often characterised by the absence of specific symptoms [6,7]. Human infections with zoonotic filariasis are reported worldwide [8] and were first described in modern research over a century ago [9]. A number of different genera of filaria have been isolated from humans, including *Dirofilaria* [10,11], *Brugia* [12,13], *Onchocerca* [14,15], *Dipetalonema* [16,17], *Loa* [18,19], and *Mansonella* [20,21]. The location of these parasites within the human body varies from species to species. Filariasis affects a range of human tissues and often evinces little or no observable host response throughout their development [22,23]. An exception occurs when the parasite enters extremely sensitive tissues, such as the conjunctiva [24].

Blood-sucking insects, such as mosquitoes, blackflies, or midges, act as biological vectors [25]. Infection is transmitted through the bite of an infected insect as it feeds upon blood [26]. Thereafter, parasite larvae mature into adult forms within the host. These adult parasites then multiply, producing microfilariae, which are subsequently ingested by blood-sucking insects along with human blood. These microfilariae undergo subsequent stages of development within the insect’s bodies [27,28,29].

Filariasis can be classified based on the location of the parasite within the host’s body: lymphatic filariasis—*Wuchereria bancrofti*, *Brugia malayi*, *Brugia timori*; subcutaneous filariasis—*Loa loa*, *Onchocerca volvulus*; and serous cavity filariasis—*Mansonella* spp. [30,31].

## 2. Lymphatic Filariasis

**Figure 1:**
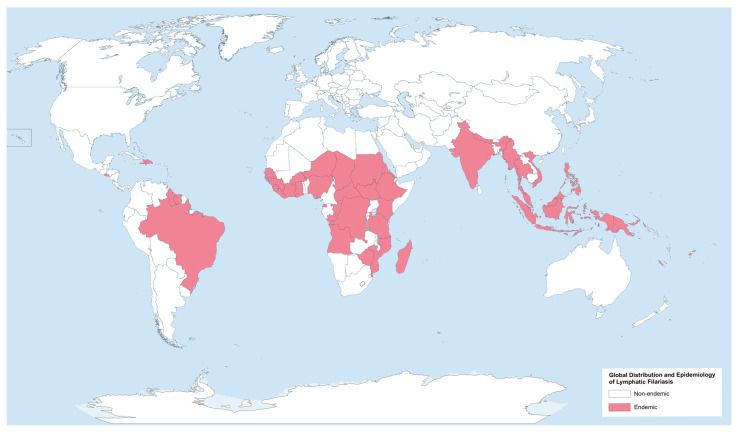
Global distribution and epidemiology of lymphatic filariasis. Created with GIMP 2.10.

The term lymphatic filariasis refers to infection with one of three nematode species: *W. bancrofti*, *B. malayi*, and *B. timori*. The majority of *W. bancrofti* infections occur in South and Southeast Asia, as well as sub-Saharan Africa. Central and South America and the Pacific islands are endemic regions. *B. malayi* has been reported in South and Southeast Asia, India, Indonesia, Thailand, Vietnam, Malaysia, and the Philippines. In contrast, *B. timori* is restricted to eastern Indonesia and East Timor (Figure 1) [32].

Lymphatic filariasis is a long-lasting, chronic, and persistent disease, with the majority of infected individuals remaining asymptomatic. However, the disease can manifest in a wide range of clinical signs, including lymphedema of the legs, lymphangitis, elephantiasis, and, uniquely in *W. bancrofti*-infected individuals, hydrocele [33]. Asymptomatic infections are characterised by the presence of several regulatory immune processes driven by living parasites that promote their long-term survival [34,35,36,37]. The precise mechanisms by which clinical disease manifests remain uncertain, although it is likely to be associated with the host’s inflammatory response to dead or dying adult parasites. The parasites’ death results in inflammation of lymphatic vessels and lymph nodes, which may cause pain and swelling in the affected area [33,38]. The susceptibility to infection, host response, and the risk of developing pathological features are familial and probably related to host genetic predisposition [39,40,41].

Lymphatic filariasis has been recognised by the World Health Organization (WHO) as a neglected tropical disease. In 2000, the Global Programme to Eliminate Lymphatic Filariasis (GPELF) was established with the objective of halting transmission through mass drug administration (MDA) of anthelmintics and alleviating the suffering of people affected by the disease through morbidity management and disability prevention (MMDP). Since the start of the GPELF, the number of infections has been reduced globally by 74% [42]. Despite the success of eradication programmes, by 2018, it was estimated that 51 million people continued to suffer from chronic symptoms of the disease, including lymphoedema, elephantiasis, and hydrocele [43]. This indicates that there is still a need for reliable and sensitive diagnostic methods to remain a priority [44].

## 3. Subcutaneous Filariasis

In the context of filariasis research, it is essential not to overlook the subcutaneous form of the disease, which presents distinct symptoms. This form is primarily characterised by cutaneous and subcutaneous symptoms resulting from nematode infections, including those caused by *O. volvulus*, *L. loa*, and *M. ozzardi* [45].

**Figure 2:**
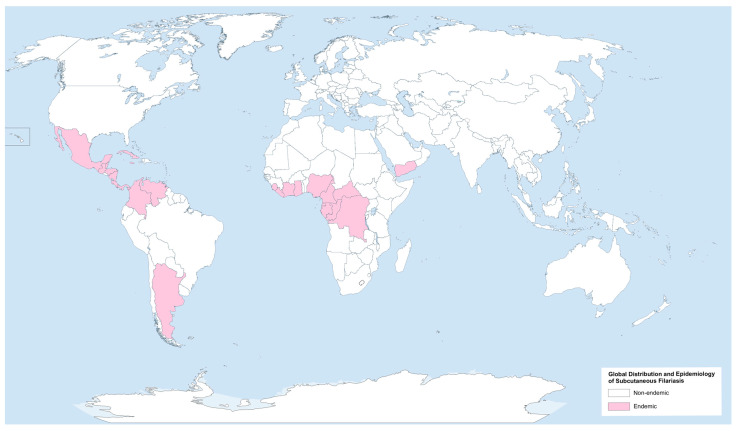
Global distribution and epidemiology of subcutaneous filariasis. Created with GIMP 2.10.

*O. volvulus* is predominantly impacting sub-Saharan Africa, Yemen, and regions of Central and South America [46,47]. *L. loa* primarily affects rural populations in Central and West Africa, with over 20 million patients chronically infected in regions with medium to high transmission rates [48], while *M. ozzardi*, one of three species in the genus *Mansonella* (the others being *M. perstans* and *M. streptocerca*) causing mansonellosis [49], is common across the neotropical region from southern Mexico to northwestern Argentina (Figure 2) [21].

The symptoms include the appearance of papules, nodules, and scratches on the skin surface, which becomes dry and thickened, often accompanied by changes in skin pigmentation and intense pruritus [50]. *L. loa* infection, also known as African eye worm or loiasis, is currently not recognised by the World Health Organisation (WHO) as one of the key neglected tropical diseases. The typical clinical manifestation of loiasis, occurring in approximately 80% of individuals, is the migration of the adult parasite under the sclera or subconjunctival eyelid tissue [51]. *M. ozzardi* infection can manifest as a skin rash, headache, fever, itching, lymphoedema (e.g., swelling of the arms or legs), and joint pain [52].

Both lymphatic filariasis and subcutaneous filariasis pose significant public health threats in endemic areas around the world. They have a crucial impact on the physical and mental health of patients and pose a challenge to health systems. To prevent further spread of the disease and to minimise the impact on affected communities, it is essential to implement comprehensive intervention in both diagnosis and treatment.

## 4. Parasitological Methods for Filariasis Diagnostics

Parasitological diagnosis is a process that involves laboratory procedures with the aim of detecting organisms in clinical specimens using morphological features and visual identification. The final identification is typically based on microscopic examination of stained slides, often utilising high-magnification techniques such as oil immersion [53]. Only a few procedures can be automated, and differences in morphological characteristics between species can be very difficult to distinguish. To become proficient in the morphological analysis of parasites using a microscope requires a considerable amount of training. Additionally, the identification of the organism is highly dependent on the skill of the person performing the examination. In order to provide accurate and effective diagnosis services in the area of medical parasitology, it is essential for laboratory professionals to possess a thorough understanding of parasite life cycles, epidemiology, invasiveness, geographic range, clinical symptoms, and the recommended therapies [54].

One of the traditional diagnostic methods for nematode infections involves the visual detection of microfilariae using stained thin and thick blood smear techniques. Morphological identification of microfilariae in blood or skin snips remains the gold standard for routine clinical diagnosis of most human filariasis. Important criteria for morphological analysis include the length and width of microfilariae and the presence or absence of a sheath [55]. The recommended staining methods for the detection of microfilariae in blood are as follows: Giemsa, Wright–Giemsa, and Delafield’s haematoxylin [54,56]. Certain parasitic nematode species exhibit periodicity, whereby microfilariae are present within the blood at specific times of day, adapted to the feeding behaviour of the vectors [57]. One disadvantage of these tests is their sensitivity, as blood sampling must be conducted at specific time intervals that vary according to the parasite species. These species include *W. bancrofti*, *Brugia* spp., and *L. loa*. *W. bancrofti* and *Brugia* species exhibit nocturnal periodicity, with the optimal time for blood sampling occurring between 10 p.m. and 2 a.m. *L. loa* exhibits diurnal periodicity, with peak microfilariae detection periods occurring between 10 a.m. and 2 p.m. [58,59].

Other parasitological methods include Knott’s technique and the membrane filtration technique, which facilitate the concentration of microfilariae present in blood samples taken from patients, enabling morphometric identification of circulating parasites. These procedures may enhance the detection of microfilariae in blood and in other bodily fluids. Knott’s technique is an easy and inexpensive technique that is more commonly used. The test procedure involves drawing approximately 1 mL of venous blood into a tube containing either EDTA or citrate. The volume of 10 mL of 10% formalin is then added to the blood. The sample is centrifuged at 300× *g* for 2 min. The supernatant is removed and the resulting precipitate is analysed under the microscope. Furthermore, the precipitate can be spread on a slide, allowed to dry, and then stained with Giemsa or haematoxylin [31,55]. The modified Knott’s test represents the most common approach. In this method, a 2% formalin is used instead of the conventional 10%. This concentration was determined to be optimal, as weaker solutions fail to form a satisfactory amount of precipitate after centrifugation, while stronger solutions precipitate haemoglobin [60]. The disadvantage of the method is the use of formaldehyde in the test, a substance contained in formalin that is mutagenic and genotoxic [61,62]. In 2012, the International Agency for Research on Cancer (IARC) classified formaldehyde as a human carcinogen [63]. Moreover, the European Chemicals Agency (ECHA) defines the substance as lethal if inhaled, toxic if swallowed and in contact with the skin, and capable of causing severe skin burns and eye damage. Formalin also requires special precautions for handling, storage, and disposal. To enhance the utilisation of the Knott test, particularly among veterinarians, research is being conducted to identify a substitute for formalin [64,65]. The membrane filtration technique involves the collection of blood in a manner comparable to the Knott test; a volume equivalent to 10 mL of 10% Teepol saline solution is added to the drawn blood. The blood–Teepol mixture is passed through a suitable filter paper (Nuclepore^®^ membrane) placed in a holder, using a syringe. Subsequently, 10 mL of water is passed through the filter, followed by 3 mL of methanol to fix the microfilariae. The filter is then placed on a slide and stained with Giemsa. The dried slide is analysed microscopically [31,55,66,67].

Skin snips, obtained by biopsy, are used to diagnose the presence of microfilariae *Onchocerca* spp. and *M. streptocerca*. For onchocerciasis, this method is considered the gold standard [68]. The sections should be thin, involving only the epidermis and superficial dermis. It is important to avoid heavy bleeding during skin biopsy to prevent contamination of the sample with peripherally circulating microfilariae. Usually, sections are taken from several sites on the body to increase the sensitivity of parasite detection. The periodicity of microfilariae in skin sections is not observed; therefore, they can be detected at any time [31,55]. This method is highly specific for active infection, whereas it is not sensitive in detecting early, light, and pre-symptomatic invasions. Furthermore, this test is not optimal for assessing the efficacy of macrofilaricidal therapies for onchocerciasis. A further limitation is the biopsy procedure, which is invasive, painful, and impractical for quick and easy diagnosis. The biopsy instruments used must be carefully disinfected between examinations to prevent the transmission of pathogens through the blood, such as HIV. The possibility of infecting subjects during biopsy collection has reduced the acceptance of skin testing among populations in endemic areas, especially in the context of countries with limited sanitation standards [69,70,71].

**Figure 3:**
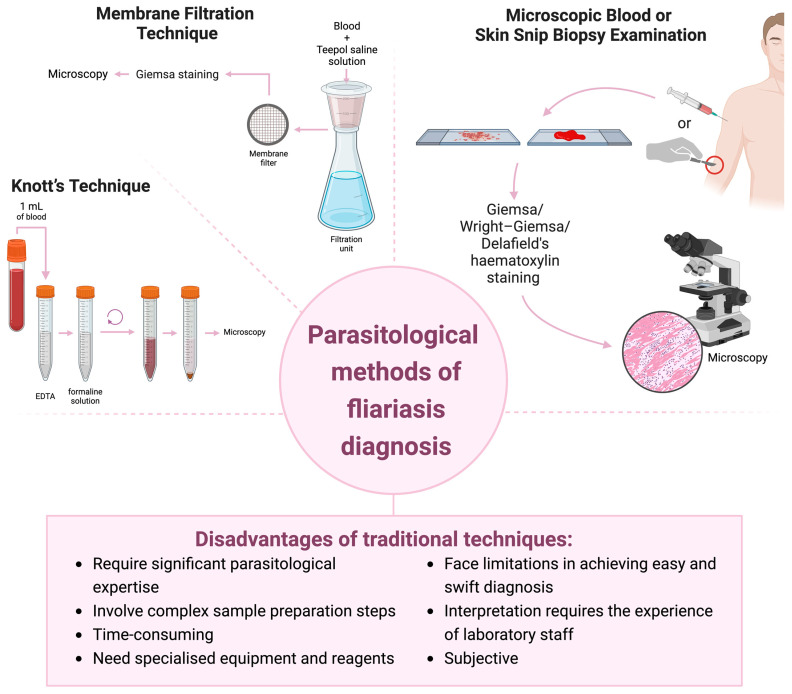
Parasitological methods for filariasis diagnostics. Created with BioRender.com.

Although traditional microscopic examination has enabled significant developments in the field of parasitology, it poses a number of problems in the diagnosis of nematode infections. Performing blood sampling at unconventional times, requiring significant parasitological expertise from microscope operators, involving complex sample preparation steps, needing specialised equipment and reagents, and facing limitations in achieving easy and swift diagnosis all contribute to the challenges in this process. Furthermore, classical parasitological methods for diagnosing the presence of microfilariae are time-consuming and subjective, as their interpretation requires the experience of laboratory staff, and the often complicated handling of samples can lead to potential errors or loss of test material. Consequently, there is a continued demand for affordable diagnostic tests that allow rapid, easy, and sensitive diagnosis of the course of nematode infections. Molecular and immunoenzymatic methods have a significant impact on the development of diagnostic techniques. Figure 3 provides a summary of the parasitological methods for filariasis diagnostics.

## 5. Next-Generation Methods for Filariasis Diagnostics

### 5.1. Brugia malayi

Although *B. malayi* accounts for only 10% of lymphatic filariasis cases, it remains a significant threat to human health [72]. Its prevalence is predominantly recorded in Southeast Asia, encompassing countries such as the Philippines, Malaysia, Indonesia, South Korea, Vietnam, and India [55]. It is estimated that more than 70% of filariasis cases in Indonesia are caused by *B. malayi* [73]. The first published case involving human infection by *Brugia* spp. was identified in 1962 in a New York City resident who presented with painless swelling of the inguinal lymph nodes [74].

There are several vectors of *B. malayi*, including mosquito species of the genus *Aedes*, *Anopheles*, and *Mansonia*, among others, depending on the geographical area [75]. The length of *B. malayi* females ranges from 43 to 55 mm, while males are shorter, reaching up to 23 mm [76]. The microfilariae of *B. malayi* are up to 230 μm long, and on Giemsa-stained preparations, their sheath is stained pink to distinguish them from the colourless sheath of *W. bancrofti* [58].

As previously stated, the most prominent clinical feature of lymphatic filariasis is the development of severe lymphoedema of the extremities (elephantiasis) and, on rare occasions, the genitalia (hydrocele), which is a consequence of vascular dysfunction [77]. Furthermore, tropical pulmonary eosinophilia, which is characterised by an eosinophilic pulmonary infiltrate, peripheral hypereosinophilia, wheezing, chest pain, splenic enlargement, and bloody sputum, is also associated with *W. bancrofti* and *B. malayi* infections [78,79,80].

#### 5.1.1. Immunoenzymatic Tests

The Brugia Rapid™ (Reszon Diagnostics International Sdn. Bhd., Selangor, Malaysia) is a rapid strip test operating on the principle of immunochromatographic technology [81]. The test is based on the specific reaction of a recombinant BmR1 protein produced in *Escherichia coli* with antiparasitic antibodies found in the patient’s serum. The antibodies react with the BmR1 antigen, which then binds to monoclonal anti-human IgG4 antibodies linked to colloidal gold [82]. The IgG4 enzyme-linked immunosorbent assay (ELISA), utilising the BmR1 antigen, demonstrated a specificity rate of 95.6% and a sensitivity rate of 96% [83]. For the Brugia Rapid™ test, the results indicated 97% sensitivity and 99% specificity [81]. Furthermore, a second validation study was conducted. The sensitivity of the strip test was found to be 93%. In the field study, compared to microscopic examination of thick blood smears, the sensitivity of the test was 87% and the specificity was 100%. The rapid strip test was therefore a useful diagnostic tool for filariasis in *B. malayi* endemic areas [84]. Other studies have also demonstrated the high sensitivity and specificity of the test in detecting infection with the parasite [85,86,87,88]. Based on the detection of specific IgG4 antibodies against recombinant BmR1 antigens, a Brugia Rapid™ test is commercially available from Reszon Diagnostics International Sdn. Bhd., Selangor, Malaysia. The WHO, in its Global Programme to Eliminate Lymphatic Filariasis, recommends the Brugia Rapid™ test for the detection of *B. malayi* invasion [89]. However, it should be noted that Brugia Rapid™ tests, which detect IgG4 antibodies specific to filaria, may generate false-positive results in patients who have been previously infected and have undergone treatment. This is because individuals affected by lymphatic filariasis may retain B-cell memory, leading to the detection of antibodies for some time after the elimination of the parasite. Currently, there is a lack of a commercial test for the detection of *B. malayi* antigens, which highlights the need for further research to improve the diagnosis of lymphatic filariasis [90].

Two-dimensional gel electrophoresis (2DE), 2DE-immunoblotting analysis of adult parasite extract, SDS-PAGE, liquid chromatography with tandem mass spectrometry (LC-MS/MS), and bioinformatics analysis enabled the identification of *B. malayi* antigens that induce the production of specific antibodies, as well as those involved in the formation of immune complexes (ICs) circulating in the blood of people with microfilaremia. This enabled the selection of 34 antibody-recognised proteins in the sera of individuals with microfilaremia who had no tissue pathology associated with parasite invasion. In addition, antigens specific to *B. malayi* microfilariae were detected, which were involved in the formation of ICs. The detection of the ICs in question may be a useful indicator for assessing the efficacy of anti-microfilariae drugs and may replace the inconvenient overnight blood sampling. These proteins have significant potential as diagnostic markers and vaccine markers. Therefore, it is crucial to continue research to gain a deeper understanding of their role in the immunopathology of filariasis and their potential therapeutic applications. Further research on these proteins may lead to the development of new diagnostic tests that can be used for the early detection of *B. malayi* and the identification of new therapeutic targets for antiparasitic drugs and potential vaccines. Furthermore, continued research on these antigens may lead to the development of new strategies to control and eradicate filariasis, which is crucial for public health, especially in endemic areas [91].

The recombinant protein BmALT-2, produced in *E. coli* strain BL21(DE3), was analysed for immunoreactivity by ELISA with human sera. This study aimed to assess the feasibility of developing a circulating filarial antigen (CFA) ALT-2 assay to detect early stages of *B. malayi* infection. The BmALT-2 protein plays an important role in filaria invasiveness and is highly expressed at the L3 stage of the parasite. The test has demonstrated high specificity, allowing further research to be conducted with the objective of developing an effective, rapid, and specific strip test to detect *B. malayi* infection [92]. In 2017, a method for the production of BmALT-2 protein in a yeast expression system was developed. The glycosylated ALT-2 protein was demonstrated to exhibit greater immunoreactivity than its non-glycosylated form, and the eukaryotic expression system based on *Pichia pastoris* yeast was identified as the optimal platform for the large-scale production of the protein [93].

Another approach to the diagnosis of *B. malayi* infection is the production of human scFv-IgG1Fc antibodies to detect recombinant BmR1 and BmSXP antigens. The recombinant BmR1 and BmSXP proteins are transcribed from the *Bm17DIII* and *BmSXP-1* genes, respectively, and are recognised by antibodies in the serum of infected patients. Both proteins are secretory products of the parasite, but their functions have not been precisely defined. In this study, three different recombinant scFv-based immunoglobulin gamma IgG1 antibodies were produced (anti-BmR1 clone 4, anti-BmXSP clone 5B, and anti-BmXSP clone 2H2). The antibodies produced were found to have high specificity and were able to bind to the corresponding proteins even at relatively low concentrations. The conjugation of Fc to scFv ensures the binding stability and solubility of scFv, and the Fc part enables the attachment of signalling molecules (e.g., probes, gold, fluorescent dyes), opening up the possibility of their use in rapid lateral flow assays. These antibodies may be employed in the development of assays for the detection of *B. malayi* antigens, particularly rapid diagnostic tests with high specificity and sensitivity. Furthermore, they can be used to avoid the detection of previous past infection with the parasite [90,94].

#### 5.1.2. Molecular Methods

A number of promising molecular techniques, including conventional polymerase chain reaction (cPCR) and real-time PCR, have been developed for the detection and differentiation of filarial nematodes, including *B. malayi* [95,96,97], loop-mediated isothermal amplification (LAMP) for the detection of *B. malayi* and *B. timori* [98], or pyrosequencing utilising SL and 5S rRNA as specific molecular markers [99]. However, highly specific and sensitive molecular tests are still required for the monitoring and detection of human infections.

In 2011, Hindson et al. developed the high-throughput droplet digital PCR (ddPCR) method. In the reaction, the mixture is split into tens of thousands of water-in-oil nanodroplets, and a single sample of genomic DNA and a subsequent primer–probe pair are subjected to simultaneous PCR amplification and quantitative evaluation of short regions of the amplicon [100]. ddPCR is a third-generation method and is more sensitive and capable of direct absolute quantitative detection than routine PCR and quantitative PCR (qPCR) [101]. Technologies have been employed to develop a duplex ddPCR assay with fluorescently labelled probes, which is capable of distinguishing and quantifying *W. bancrofti* and *B. malayi* in mosquito and mammalian blood samples and also detecting co-invasion [102]. The authors observed that the method is straightforward to perform and interpret, relatively inexpensive, and provides high specificity and sensitivity in detecting *W. bancrofti* (100% and 100%) and *B. malayi* DNA (98.3% and 100%).

In recent years, high-resolution melting (HRM)-PCR has become a widely used method in parasitological diagnostics [103,104,105]. In this method, products undergo thermal denaturation after the real-time PCR. Fluorescence changes caused by the release of intercalating dye from the DNA strand are monitored in real time. By comparing the resulting melting curves of unknown samples with the melting curve profiles of known isolates, it is possible to assign them to specific strains or species [106]. A real-time HRM PCR assay was developed for the detection and differentiation of *W. bancrofti*, *B. malayi*, *D. immitis*, and *B. pahangi* in vectors and blood samples. A single primer pair that is employed to amplify the arrays of all four species enables the detection of the 5S rRNA gene and spliced leader sequences [103]. Identification of each species is possible based on different melting temperatures [107]. In terms of cost per sample, the method is less expensive than multiplex real-time PCR methods. Furthermore, assays can be performed on a large number of samples simultaneously, with only very small sample volumes needed for a single analysis [108]. Additionally, reactions can be performed on paraffin-embedded samples, thus providing an effective alternative diagnostic procedure [109].

#### 5.1.3. Other Tests

Since the 1990s, a number of diagnostic techniques, such as the identification of specific antibodies and circulating antigens, and PCR protocols have been introduced to improve the detection of filariasis and increase the reliability of the results [110,111]. However, none of these methods is sensitive and precise enough to simultaneously identify all three aetiological factors of lymphatic filariasis. Consequently, it is relatively simple for the clinician to make an erroneous diagnosis, particularly when the patient’s symptoms are atypical, especially in non-endemic areas. Although shotgun metagenomics has been extensively utilised for virus identification [112,113] and the detection of bacterial pathogens [114], its application in parasitic infections remains limited [115]. In the case of *B. malayi*, a case was described of a 34-year-old Chinese student who, despite repeated visits to hospitals in both the US and China, could not obtain a definitive diagnosis [116]. The patient exhibited no overt symptoms of lymphatic filariasis [117]. However, testing for filariasis was conducted via PCR of the conserved genes *B. malayi*, *B. timori*, and *W. bancrofti*. The results of the analysis demonstrated the absence of parasites in the patient’s blood. Given the absence of a diagnosis and the patient’s increased itching and swelling of the body, a metagenomic approach was finally applied in order to identify the pathogen. The results of this analysis indicated that the patient was infected with *B. malayi*. This provides hope for the diagnostic identification of not only typical lymphatic filariasis infections but also those that are cryptic [116].

Table 1 presents a comparative analysis of diagnostic methods for *B. malayi* infection, as previously outlined in the preceding sections.

### 5.2. Onchocerca volvulus

Onchocerciasis in humans, primarily caused by *O. volvulus*, remains a significant neglected tropical disease, continuing to exhibit a high incidence and contributing to poverty in endemic areas [118]. As indicated by estimates from the Global Burden of Disease Study 2015, approximately 15.5 million people are currently living with onchocerciasis, including 12.2 million people with onchocerca skin disease (OSD) and 1.025 million people with vision loss (known as river blindness) [119].

This nematode is transmitted by the female whiteflies of the genus *Simulium* [120]. In the human host, the worms reach maturity after 1–3 years, and the adult worms (macrofilariae) reside in nodules under the skin, where females can live for up to 15 years. During this period, they are capable of producing millions of microfilariae that migrate under the skin of infected individuals. The males of *O. volvulus* have a length of 23 mm and a posterior end which curves ventrally. In contrast, females have a length ranging from 230 to 700 mm [121]. 

The most prominent clinical signs in individuals with onchocerciasis are skin lesions, including pruritic dermatitis and nodules [122]. Many patients suffer from visual disturbances commonly referred to as river blindness [123,124]. Additionally, disease has been linked to epilepsy, specifically onchocerciasis-associated epilepsy (OAE) [125,126] and nodding syndrome [127].

#### 5.2.1. Immunoenzymatic Tests

The immunoenzymatic techniques used in the detection of *O. volvulus* have been extensively researched, which clearly demonstrates their significance. Despite the existing knowledge, there are ongoing endeavours to refine the existing tests and identify new diagnostic procedures. This continuous effort is understandable, as improvements in diagnostic efficiency and accuracy can be crucial for the treatment and control of onchocerciasis.

The OV-16 antibody test is the only WHO-approved method for the diagnosis of *O. volvulus* infection in humans [128]. The OV-16 ELISA test is based on the detection of antibodies that are specific to the recombinant OV-16 *O. volvulus* antigen. The assay was developed in the 1990s [129]. It has been observed that approximately 15–25% of individuals have genetic alterations that prevent the induction of an immune response to the OV-16 antigen [130]. Furthermore, this test is unable to distinguish between past and active infections, as parasitic antibody levels remain high even after the disease has ended [70]. Although the detection of anti-Ov16 IgG4 immunoglobulins is more sensitive and less invasive than microscopic analysis of skin biopsies [131], frequent false-negative results give a false sense of security and make it difficult for clinicians to make a diagnosis. 

The OV-16 ELISA, developed by Cama et al. (2018), represents a modification of a protocol utilised by multiple laboratories engaged in evaluating the effectiveness of an onchocerciasis elimination programme in Guatemala [132,133]. The modifications involved a reduction in antigen concentration from 1.0 to 0.5 μg/well, an increase in incubation temperature from room temperature to 37 °C, and an optimisation of the reaction time of chromogenic substrate at four different times (15, 30, 45, and 60 min). The utilisation of specific incubation temperatures and a fixed time of 60 min for the reactivity of the chromogenic substrate proved to be significant factors in enhancing the accuracy of this assay. The specificity and sensitivity of the modified Ov16 IgG4 ELISA were reported to be 99.7% and 88.23%, respectively [134].

Additionally, there is a growing body of research aimed at the identification of new antigens that may enable more specific and sensitive diagnoses of *O. volvulus* infection. In 2020, Shintouo et al. designed and validated the recombinant chimeric antigen OvMANE1. An indirect ELISA was performed to quantify specific IgG antibodies present in the sera of infected and uninfected individuals. Furthermore, the OvMANE1 antigen did not cross-react with antibodies in sera collected from patients infected with related nematodes. Studies have demonstrated the considerable potential of OvMANE1 for use in the development of antigenic diagnostic tests and antibody capture reactions, which are essential for monitoring the progress of onchocerciasis eradication programmes [135]. Additionally, novel potential diagnostic peptides were produced and characterised, including OvNMP-48 [136]; OvMP-1, OvMP-2, OvMP-3, OvMP-23 [137]; OvHSP70 [138]; Ov58GPCR-ECD [139]; OVOC10469, OVOC3261 [140]; OV-17 and OV-33 [141]. These studies provide an important foundation for further attempts to use the identified proteins as specific biomarkers in the diagnosis of human onchocerciasis.

Another diagnostic approach is the use of recombinant antibodies as controls in serological tests. Among others, a recombinant human anti-Ov16 IgG4 clone has been identified and used to produce standards for the serological OV-16 ELISA. This provides a consistent and standardised control between tests. The anti-Ov16 IgG4 control can facilitate ELISA plate validation and normalisation of data for analysis and comparison. Furthermore, the utilisation of a singular positive control may facilitate comparisons of assay performance with novel rapid diagnostic tools, such as the Bioline^®^ ONCHOCERCIASIS IgG4 rapid diagnostic assay, which allows for the significant characterisation of the limits of detection of specific antibodies [142].

The Bioline^®^ ONCHOCERCIASIS IgG4 rapid diagnostic test (OV-16 RDT), manufactured by Abbot (Lake Forest, IL, USA), is a commercial, qualitative, rapid immunochromatographic test that detects IgG4 antibodies against the *O. volvulus* specific OV-16 antigen from patient blood [143]. The test was compared with the OV-16 ELISA and microscopic detection of microfilariae in skin sections. The OV-16 ELISA demonstrated a higher sensitivity of 83%, while the OV-16 RDT achieved a value of 74.8% and the examination of skin sections showed the lowest sensitivity of 71.4%. Moreover, the OV-16 RDT exhibited a greater degree of specificity (98.6%) compared to the OV-16 ELISA (84.8%) [144]. In other studies, the OV-16 RDT and OV-16 ELISA were observed to have a satisfactory percentage agreement of 99.2% in positive samples, identifying parasitic invasion. Furthermore, it was demonstrated that the OV-16 RDT could detect fewer individuals with *O. volvulus* infection than the OV-16 ELISA [145].

#### 5.2.2. Molecular Methods

As with the detection of other nematodes causing filariasis, PCR can also be used to identify *O. volvulus*. These methods rely on the amplification of a repetitive 150 nucleotide sequence (designated O-150) and its subsequent detection with, for instance, a specific probe utilising the Southern blot technique [146,147,148]. In 2011, Fink et al. described a melting curve-based qPCR using degenerate primers to detect a 154 bp long O-150 repeat amplicon (O-150 qPCR) [149]. However, it has been observed that the long amplicon results in limited sensitivity, and the absence of a hybridisation probe may reduce the specificity of the assays [150]. Accordingly, Mekonnen et al. introduced a methodology utilizing a hydrolysis probe-based qPCR technique targeting the 5S rRNA gene of *O. volvulus* (O-5S qPCR), with the aim of refinement and optimization [151]. The new method was found to increase the detection limit by half compared to O-150 qPCR. The sensitivity of the assay was enhanced by the use of a highly stable hydrolysis probe that is 100% specific for *O. volvulus* [150].

A one-step method for detecting *O. volvulus* infection in skin scrapings based on the length polymorphism of the first internal transcribed spacer (ITS1) repeat of ribosomal DNA has also been described [152]. These regions are used to detect and differentiate filarial parasite species [153,154]. This approach allows the development of a single set of ‘universal’ primers that can amplify the DNA of various filarial species, and then differentiate them based on amplicon length or sequence polymorphism. The authors adapted this method to real-time polymerase chain reaction with melting curve analysis (qPCR-MCA), which allows the detection and differentiation of PCR products of different sizes in a single tube and during a single PCR. Limiting the assay to a single tube reduces the risk of error due to carryover or cross-contamination [152]. The qPCR-MCA assay was compared with the qPCR-O-150 and the O-150-PCR ELISA [155]. A total of 471 skin biopsies were analysed, with 47.5%, 43.5%, and 27.0% of them found to be positive for *O. volvulus* in the qPCR-O-150, qPCR-MCA, and O-150-PCR ELISA tests, respectively. A comparison of the sensitivity and specificity of the qPCR-MCA test with the qPCR-O-150 test as a comparator demonstrated values of 89.3% and 98.0%, respectively, while for the O-150-PCR ELISA, the values were 56.7% and 100%. The majority of qPCR-MCA misclassifications occurred in mixed infections, which highlights the need for specific qPCR tests.

LAMP was first described in 2000 [156]. LAMP is an alternative to cPCR methods that is characterised by ease of execution and the possibility of colorimetric readout [157]. This technique is particularly useful in resource-limited settings, where it does not require expensive equipment [158,159], which is usually not available in endemic locations, making diagnosis difficult. Additionally, LAMP is less sensitive to the inhibitors present in clinical samples and insects, which can inhibit Taq polymerases used in PCR. The technique relies on DNA synthesis, with automatic cyclic strand displacement guided by *Bst* DNA polymerase. The reaction proceeds without denaturation of the DNA template [160], thus allowing it to be carried out at isothermal temperatures. Using the reaction carried out, the matrix can be amplified up to 10^9^–10^10^ times in 15 to 60 min. The amplified products consist of a series of parent DNA loops of different lengths [161]. The results are read by visual inspection of the turbidity due to precipitation of white magnesium pyrophosphate, which is a by-product of DNA synthesis [162], or by visual and UV inspection of DNA amplification using a fluorescent dye [163]. This method has also been applied in the diagnosis of *O. volvulus* infection. *COX1* sequences are amplified, utilizing DNA extracted from skin biopsies as the template, a material concurrently employed in the PCR O-150 procedure [164,165,166]. The LAMP test shows high specificity for the genus *Onchocerca*. However, it has been observed that the test can also detect other species, including *Onchocerca ochengi* [167]. Nevertheless, this is considered a significant problem because the *O. ochengi* is typically found in cattle, not in humans, and therefore, its presence in the biopsy is negligible [168]. The molecular testing still depends on the detection of *O. volvulus* DNA in the skin and therefore may not be reliable after treatment with antiparasitic drugs. Skin biopsies are also invasive and are becoming less common, with endemic communities often refusing the procedure [169].

An alternative approach is the detection of circulating DNA and microRNA (miRNA) of *O. volvulus*, where sampling is less invasive and diagnosis is not based on the temporal presence of microfilaremia [170]. miRNAs are short (18–22 nucleotides), single-stranded, non-coding RNAs responsible for regulating mRNA expression in both animal and plant cells [171]. The literature indicates that miRNAs may play a significant role in the parasite’s adaptation to the host [172] as well as in modulating its innate immunity [173]. A number of attempts have been made to utilise circulating, body fluid-derived miRNAs and DNA as a diagnostic tool. miRNAs belonging to *O. volvulus* have been identified in the serum of infected individuals [174,175]. Unfortunately, the majority of studies indicate that they are not a sufficient diagnostic tool or biomarkers of infection [170,176,177].

#### 5.2.3. Other Tests

With advances in technology and science, novel diagnostic methods for *O. volvulus* are emerging that transcend traditional approaches based on immunoenzymatic and PCR techniques. One such approach is the detection of N-acetyltyramine-O,β-glucuronide (NATOG) in urine, which has emerged as a highly promising biomarker specific for *O. volvulus* [178]. NATOG is a neurotransmitter-derived secretory molecule from *O. volvulus* [179]. The potential of NATOG as a diagnostic marker was investigated by assessing its stability under conditions resembling those in tropical environments and its adsorption characteristics. A further study was conducted to evaluate the use of lateral flow circulation (LC) with fluorescence to detect NATOG in urine and to analyse its levels in samples from individuals with nodules present but with no or very low microfilariae. The results were then compared with control groups to assess the diagnostic performance of this biomarker. The evaluation of the use of NATOG in rural areas in sub-Saharan Africa demonstrated that it retained its stability even after 24 h of incubation at 50 °C and exposure to sunlight for 36 h. Despite maintaining relatively high stability, the sensitivity of NATOG was found to be relatively low in patients with very low microfilaria levels in the skin. These findings indicate that NATOG is most likely to be most effective as a biomarker in cases of very advanced *O. volvulus* infections, where the parasite titre in the body is high [178].

Table 2 presents a comparative analysis of diagnostic methods for *O. volvulus* infection, as previously outlined in the preceding sections.

### 5.3. Onchocerca lupi

In 1967, a previously unidentified *Onchocerca* spp. was discovered in wolves (*Canis lupus*) as *O. lupi* [180]. Studies have demonstrated the distinctive morphology of this species, as well as the fact that its 5Sr DNA spacer sequences do not resemble those of other nematodes of the genus *Onchocerca* [181]. *O. lupi* is typically associated with the disease onchocerciasis, which primarily affects dogs [182,183] and cats [184]. However, there have been an increasing number of reports of human infection with this parasite in various geographical locations around the world [185,186,187]. Adult male *O. lupi* is white in colour and measures between 43 and 50 mm in length, with a diameter of approximately 0.15 mm [182]. In contrast, it is challenging to ascertain the length of the female, as it is difficult to remove it entirely from the nodules. However, the longest fragments were reported to be between 100 and 165 mm [180,188].

The clinical signs observed in humans are highly variable and may include the formation of nodules on the conjunctiva occurring together with inflammation of varying degrees in the ocular region [186,187]. Additionally, the development of soft masses within the mid-ciliary canal, is observed in this species, which is a unique characteristic [189,190].

#### 5.3.1. Immunoenzymatic Tests

The diagnosis of *O. lupi* infection is based on the detection of microfilariae within skin biopsies and the identification of ocular nodules located on the eyelids, conjunctiva, or sclera. 

In particular, skin biopsy is an invasive and time-intensive procedure, thus necessitating the exploration of novel diagnostic modalities. Latrofa et al. (2021) employed an immunoproteomic approach, integrating immunoblotting and mass spectrometry, to identify novel antigens that could be utilised in diagnostic tests. This study indicates that Ol-MJA and Ol-PARA, two linear protein peptides, may be suitable as promising candidates as biomarkers for diagnostic test development. Furthermore, Ol-MJA and Ol-PARA have been recognised by IgG antibodies in the sera of dogs infected with *O. lupi* [191]. In 2024, an indirect ELISA was conducted to evaluate the specificity and sensitivity of parasite detection using the previously characterised linear peptides of the Ol-MJA and Ol-PAR proteins, in addition to three newly identified peptides from Ol-MJA. The proposed assay based on the linear peptides of the Ol-MJA and Ol-PAR proteins demonstrated high immunoreactivity in all assays, including sera from dogs with low levels of *O. lupi* microfilaremia. Furthermore, the indirect ELISA exhibited high accuracy, with 100% specificity and sensitivity, ranging from 85.45% to 94.55%, depending on the peptide tested. Although *O. lupi* predominantly causes onchocerciasis in dogs and cats, the number of human infections is increasing. Therefore, the development of a non-invasive, rapid, specific, and sensitive tool for serodiagnosis of *O. lupi* infection is greatly needed, both for monitoring epidemiology and helping sick people and animals. Further research should concentrate on assessing the efficacy of the test in detecting *O. lupi* infection in cats and humans and improving this diagnostic method [192].

#### 5.3.2. Molecular Methods

Mitochondrial markers have gained significant recognition in phylogenetic and epidemiological studies due to their clonality, the rare phenomenon of recombination, and their faster evolution than nuclear genes. This makes their analysis possible, enabling the distinction of closely related individuals from different geographical locations and the reconstruction of their phylogeny [193,194]. In individuals of the genus *Onchocerca*, the mitochondrial genes for NADH dehydrogenase subunit 5 (*ND5*) and cytochrome c oxidase subunit 1 (*COX1*) are amplified and sequenced [186,195,196,197]. Nevertheless, this method requires a considerable investment of labour and may be less sensitive than desired, particularly in the identification of microfilariae. This limits the scope of extensive epidemiological investigations which involve both vertebrates and potential vectors.

In 2018, a paper by Latrofa et al. was published in which a quantitative hybridisation probe-based qPCR assay was developed to detect *O. lupi* DNA in canine and feline samples and putative vectors. The selected target gene was *COX1*, which is considered to be a specific ‘barcode’ of filarial nematodes [198,199]. This gene has been demonstrated to have a high amplification efficiency and a high copy number, allowing the detection of minimal amounts of DNA [200]. These facts, combined with a skilfully designed probe that was 100% specific for *O. lupi* DNA, allowed the development of a reliable method. The assay is characterised by high sensitivity, enabling the detection of small quantities of DNA, coupled with high efficacy. The analytical specificity and sensitivity of the developed method were evaluated by examining genomic DNA from skin samples with different parasite numbers, through serial dilutions of microfilariae DNA and adult *O. lupi* specimens. The qPCR test was also highly specific in detecting *O. lupi* DNA in both co-infected samples from dogs and potential vector species. This obviates the need to verify the test result with the sequencing required by cPCR methods [201].

Despite obtaining rather impressive results, the above method was further modified, this time using human samples. The assay was modified to include primers designed for the genus *Onchocerca*, as well as two species-specific (*O. lupi* and *O. volvulus*) TaqMan probes targeting the *COX1* locus. This allowed for simultaneous detection and differentiation of *O. lupi* and *O. volvulus*. The newly developed qPCR method demonstrated high efficiency, with satisfactory limit of detection and no cross-reactivity with human DNA or other parasites tested. The results were consistent with those obtained using 12S and 16S PCR, confirming a 95% (20/21) agreement with microscopic analysis. It is noteworthy that the novel qPCR method was unable to detect a single case of *O. lupi* that was positive under the microscope. However, the authors hypothesise that the problem may not have originated in the method itself but rather in a too low concentration of total DNA, resulting from an initially very small clinical sample. The analytical sensitivity of this reaction was comparable to that reported by Latrofa et al. (2018) [202].

The qPCR method allowed for the specific identification of *O. lupi* and *O. volvulus* parasites, even in cases where sequencing data were incomplete or absent, or where identification by microscopy was limited to genus determination only. Nevertheless, it is important to acknowledge that the efficacy of PCR assays could be influenced by a multitude of variables, including suboptimal sample storage and fixation, which can result in inaccurate DNA amplification [203].

Table 3 presents a comparative analysis of diagnostic methods for *O. lupi* infection, as previously outlined in the preceding sections.

### 5.4. Wuchereria bancrofti

Among the three human parasites responsible for lymphatic filariasis, *W. bancrofti* is the most prevalent. This cosmopolitan parasite is found in tropical, subtropical, and temperate regions, including South Asia, East Asia, Africa, the Western Pacific, and, to a lesser extent, certain locations in the Americas [204].

The life cycle of *W. bancrofti* is similar to other lymphatic filariasis-causing parasites. During a blood meal, an infected female mosquito introduces third-stage larvae of filariasis onto the skin of a human host, where they penetrate the bite wound. These larvae mature into adults, primarily inhabiting the lymphatic system. Females measure 80 to 100 mm in length, while males are smaller, measuring approximately 40 mm. Adult parasites produce microfilariae that are sheltered and exhibit higher nocturnal activity. These microfilariae migrate through the lymphatic and blood channels, actively traversing through these bodily fluids. The cycle then closes when the mosquito takes up microfilariae from the blood during a meal [77].

The invasion caused by *W. bancrofti*, although rarely leading to death, is considered a major cause of disability, permanent disability, and chronic morbidity [205].

#### 5.4.1. Immunoenzymatic Tests

BinaxNOW^®^ (Alere, Scarborough, ME, USA) is a commercial lateral flow assay (LFA) that was developed in the early 2000s. However, it has been observed that the test has low sensitivity and stability, a short shelf life (3 months), variable performance which significantly affects the results obtained, requires cold storage until use, and has a relatively high cost. Another issue with the test is the narrow time window for reading the test results. The manufacturer’s instructions recommend reading the test after 10 min, and delaying this reading (e.g., after 20 min) often leads to false-positive results. Furthermore, cross-reactivity of the test with phyla other than *W. bancrofti* has been reported [206,207,208,209,210,211].

Another commercially available test that has replaced BinaxNOW^®^ is the Alere Filariasis Test Strip (Alere, Scarborough, ME, USA). It was launched in 2013 and is recommended by the WHO for the diagnosis of filariasis caused by *W. bancrofti* in endemic areas [212]. Additionally, the STANDARD Q Filariasis Antigen test (SD Biosensor, Suwon, South Korea) is also commercially available [213]. All point-of-care (POC) tests discussed are based on the technology of detecting circulating antigens (CFA) of *W. bancrofti* in the patient’s blood. Antigen tests are more sensitive for filariasis infection than tests detecting microfilariae in blood collected at night (nocturnal periodicity of microfilariae in blood) [214]. The current antigen tests are less expensive, have a longer shelf life and higher analytical sensitivity compared to the previously used BinaxNOW test^®^ [215,216].

BLF Rapid™ (Universiti Sains Malaysia, Gelugor, Malaysia) is a prototype immunochromatographic assay based on the detection of antiparasitic IgG4 antibodies against the recombinant BmSXP protein in sera from patients with filariasis caused by *W. bancrofti*. Independent evaluations have demonstrated a diagnostic sensitivity of 84% and specificity of 100%, indicating the test’s potential as a diagnostic tool [217].

A recombinant monoclonal antibody (5B) specific to the BmSXP [218] antigen was utilised in the development of an ELISA. This enabled the detection of circulating parasite antigens in the serum of patients infected with *W. bancrofti*. The diagnostic specificity and specificity of the test were demonstrated to be 100% when tested with sera from healthy individuals and patients with other parasitic diseases. Consequently, the test has the potential to be a promising alternative test for the detection of the *W. bancrofti* antigen [219].

The recombinant protein Wb-Bhp-1, which is a homologue of the BmR1 protein used in tests to detect *B. malayi*, was evaluated for the diagnosis of *W. bancrofti* infection. The ELISA demonstrated variable sensitivity for the detection of microfilariae, which differed between sera from patients from different countries. Furthermore, low cross-reactivity was observed with samples from individuals with onchocerciasis or loiasis, which is necessary for the test to be useful for lymphatic filariasis serology in many areas of sub-Saharan Africa [220].

The recombinant protein HSP70 (BmHSP70) of *B. malayi* was characterised for its ability to diagnose the asymptomatic stage of *W. bancrofti* microfilariae infection. Based on ELISA, it was concluded that the antigen tested, in terms of sensitivity and specificity, could be used to diagnose the early stage of microfilariae infection [221].

The immunogenicity of recombinant *B. malayi* (Bm-TPP and BmAF-Myo) and *Wolbachia* (Wol Tl IF-1, wBm-LigA) proteins were analysed by immunoblotting techniques and the measurement of specific IgG antibodies by ELISA. The study included patient serum samples from the three main endemic zones of filariasis caused by *W. bancrofti*: endemic normal (EN), microfilariae carriers (MF), and chronic filarial patients (CP). The results of the analysis demonstrated that individuals from the endemic area (EN) of *W. bancrofti* had specific IgG antibodies directed against all four *B. malayi* and *Wolbachia* proteins tested. The strong reactivity of EN sera to all tested proteins suggests their potential immunoprotective nature, thereby indicating the potential use of recombinant *B. malayi* proteins as diagnostic markers or vaccine candidates against *W. bancrofti* [222].

#### 5.4.2. Molecular Methods

Amplification-based molecular methods, such as PCR, have been demonstrated to be highly sensitive and specific for the detection of *W. bancrofti* microfilariae [223,224,225]. However, they are not routinely used due to the high cost and complexity of the procedures, which require trained personnel and sophisticated equipment that is difficult to access in endemic areas [226].

A potential solution is the utilisation of compact PCR-based methodologies and devices that are capable of detecting parasites at the site of patient sampling [227,228]. The method utilises a miniPCR^®^ thermocycler (DBA miniPCR bio; Amplyus LLC., Cambridge, MA, USA), which is an inexpensive and portable device. However, this method requires electrophoretic analysis, which is not possible to perform under field conditions. In order to circumvent these steps, a combination of miniPCR and duplex lateral flow dipstick (DLFD) was selected for the rapid detection of *W. bancrofti* (and *B. malayi*) in human blood. In the laboratory evaluation, the miniPCR-DLFD and the stained blood smear, considered the gold standard for detecting MF, showed concordant results, confirming the efficacy of the miniPCR-DLFD test in the diagnosis of lymphatic filariasis. The detection limit of the miniPCR-DLFD was 4 mg of *W. bancrofti* per ml of blood. Furthermore, no cross-reactivity was observed when tested with the other 11 parasites. The only identified limitation observed was the *HhaI* amplicons of *B. timori*, which demonstrated 96.77% identity with *B. malayi*. However, this does not impede the identification of *W. bancrofti*, and furthermore, the endemic region of *B. timori* is confined to East Timor and a few islands in eastern Indonesia only [229].

As previously stated in the context of *O. volvulus* detection, the LAMP assay also demonstrates significant advantages over cPCR. The reactions can be carried out using a straightforward apparatus, and the visualisation of amplicons is achieved through the use of pH-sensitive dyes or by observing the turbidity of the reaction mixture [162,230]. An additional advantage is that the lyophilised reagents do not require refrigeration, which ensures that they remain stable even at high temperatures. The LAMP technique is also used to identify *W. bancrofti* infestations. The LAMP assay, developed by Kinyatta (2021), demonstrates that this method can be both highly specific (97.3%) and sensitive (the lowest concentration detected was 10^−6^, corresponding to 1 microfilariae per 200 µL). Moreover, the LAMP reaction has the advantage of greater specificity compared to cPCR methods due to the use of four to six primers (FIP, BIP, F3, B3, and/or 2 loop primers) to recognise six to eight different regions of interest in a sequence [231].

Table 4 presents a comparative analysis of diagnostic methods for *W. bancrofti* infection, as previously outlined in the preceding sections.

## 6. Conclusions

Filariasis, caused by nematodes, remains a formidable challenge to global public health. Despite concerted efforts and international initiatives aimed at eradicating the disease, definitively halting its proliferation has remained elusive. A significant factor contributing to this persistence is the limited understanding of the intricate mechanisms by which parasites evade detection by the host immune system. Over the last several decades, molecular biology methods have developed rapidly, opening up new possibilities in the study of filariasis and other parasitic diseases. The introduction of modern DNA sequencing techniques, such as next-generation sequencing (NGS), has enabled in-depth analyses of parasite genomes and their interactions with the host. Furthermore, PCR techniques allow rapid and sensitive detection of parasite genetic material even in small biological samples, which contributes to rapid disease diagnosis and surveillance. Molecular diagnostics plays a pivotal role in comprehending the intricate adaptive mechanisms of parasites and in developing more efficient methods for detecting and treating filariasis. Application of molecular techniques enables the identification of genetic markers of parasites and the analysis of their interactions with the host immune system. Moreover, molecular biology techniques not only facilitate the delineation of parasite genes but also enable the production of recombinant proteins with potential utility as diagnostic biomarkers or therapeutic targets. ELISA tests based on recombinant proteins enable more accurate and specific detection of antibodies against parasites, which can be crucial for rapid diagnosis and disease monitoring. By combining these advanced technologies, researchers aspire to devise comprehensive strategies for combating filariasis that are both efficacious and precise. Ongoing research endeavours aim to refine diagnostic tools and pioneer innovative therapies, thereby fortifying global efforts to safeguard the health of populations worldwide against this formidable parasitic threat.

## Figures and Tables

**Table 1:** Immunoenzymatic, molecular, and other tests for the detection of *B. malayi* infection.

Type of Method	Method	Description	Article
Immunoenzymatic	Brugia Rapid™	Rapid diagnostic test utilising a recombinant BmR1 protein	[81,82]
	Immunoproteomic	Identification of antigens with potential diagnostic applications	[91]
	ELISA	Utilises recombinant BmR1 antigen	[83]
		Utilises recombinant BmALT-2 antigen	[92,93]
		Utilises human scFv-IgG1Fc antibodies to detect recombinant BmR1 and BmSXP antigens	[90,94]
Molecular	Duplex ddPCR	Amplification of HhaI repeat element	[102]
	Real-Time HRM-PCR	Amplification of 5S rRNA gene and spliced leader sequence SL1	[103]
Other	Metagenomic	Massively parallel sequencing	[116]

**Table 2:** Immunoenzymatic, molecular, and other tests for the detection of *O. volvulus* infection.

Type of Method	Method	Description	Article
Immunoenzymatic	OV-16 ELISA	Detection of antibodies specific to the recombinant OV-16 *O. volvulus*	[129]
	Modified OV-16 ELISA	Enhanced OV-16 ELISA with improved accuracy	[134]
	ELISA	Utilises recombinant chimeric antigen OvMANE1	[135]
		Utilises recombinant human anti-O16 IgG4 as a control	[142]
	Bioline^®^ ONCHOCERCIASIS IgG4 (OV-16 RDT)	Rapid diagnostic test for the detection of anti-O16 IgG4 *O. volvulus*	[144,145]
Molecular	PCR	Amplification of a repetitive sequence (O-150)	[146,147,148]
	O-150 qPCR		[149]
	O-5S qPCR	Amplification of intergenic spacer region of the 5S rRNA	[151]
	qPCR-MCA	Amplification of ITS1 region	[152]
	LAMP	Amplification of *COX1* gene fragment	[167]
	Circulating DNA, miRNA profile analysis	Insufficient diagnostic tool	[170,176,177]
Other	LC-MS/MS	Detection of N-acetyltyramine-O, β-glucuronide in urine	[178]

**Table 3:** Immunoenzymatic, molecular, and other tests for the detection of *O. lupi* infection.

Type of Method	Method	Description	Article
Immunoenzymatic	Immunoproteomic	Integrates immunoblotting and mass spectrometry to identify novel peptides for diagnostic tests	[191]
	ELISA	Utilises linear peptides of the Ol-MJA and Ol-PAR proteins	[192]
Molecular	qPCR	Amplification of *COX1* gene fragment	[199,202]

**Table 4:** Immunoenzymatic, molecular, and other tests for the detection of *W. bancrofti* infection.

Type of Method	Method	Description	Article
Immunoenzymatic	BinaxNOW^®^	Rapid diagnostic tests	[207]
	Alere Filariasis Test Strip		[212]
	STANDARD Q Filariasis Antigen Test		[213]
	BLF Rapid™		[217]
	ELISA	Utilises recombinant monoclonal antibody (5B) specific to the BmSXP	[218,219]
		Utilises recombinant Wb-Bhp-1 antigen	[220]
		Utilises recombinant BmHSP70 antigen	[221]
		Utilises recombinant Bm-TPPBmAF-Myo, Wol Tl IF-1, wBm-LigA antigens	[222]
Molecular	miniPCR-DLFD	Amplification of 181 bp region of *Ssp*I repetitive noncoding DNA sequence	[229]
	LAMP	Amplification of 18S rRNA fragment	[231]

## Data Availability

Not applicable.
